# Sacral Fracture Nonunion Treated by Bone Grafting through a Posterior Approach

**DOI:** 10.1155/2013/932521

**Published:** 2013-04-30

**Authors:** Sang Yang Lee, Takahiro Niikura, Yoshitada Sakai, Masahiko Miwa, Kotaro Nishida, Ryosuke Kuroda, Masahiro Kurosaka

**Affiliations:** Department of Orthopaedic Surgery, Kobe University Graduate School of Medicine, 7-5-1, Kusunoki-cho, Chuo-ku, Kobe 650-0017, Japan

## Abstract

Nonunion of a sacral fracture is a rare but serious clinical condition which can cause severe chronic pain, discomfort while sitting, and significant restriction of the level of activities. Fracture nonunions reportedly occur most often after nonoperative initial treatment or inappropriate operative treatment. We report a case of fracture nonunion of the sacrum and pubic rami that resulted from non-operative initial treatment, which was treated successfully using bone grafting through a posterior approach and CT-guided percutaneous iliosacral screw fixation combined with anterior external fixation. Although autologous bone grafting has been the gold standard for the treatment of pelvic fracture nonunions, little has been written describing the approach. We utilized a posterior approach for bone grafting, which could allow direct visualization of the nonunion site and preclude nerve root injury. By this procedure, we were able to obtain the healing of fracture nonunion, leading to pain relief and functional recovery.

## 1. Introduction

Great progress in trauma care systems during the past decade has resulted in an increased survival rate of injured patients with severe pelvic injuries [1–3]. Although posttraumatic pelvic fracture nonunion is thought of as uncommon, their presence contributes to a poor functional outcome [4]. In fracture nonunion cases, chronic pain is often the main complaint (especially during weight-bear) along with significant restriction in the level of activities. Fracture nonunions reportedly occur most often after non-operative initial treatment or inappropriate operative treatment (such as use of an external fixator as the definitive treatment for unstable pelvic ring fractures) [5–11]. Surgical treatment of pelvic fracture nonunions is technically demanding and has potentially serious complications such as nerve or vascular injuries, blood loss, and infection [6]. Pelvic nonunion is usually treated with open reduction and internal fixation, excision of scar tissue, and autologous bone grafting [6, 9, 12–15]. However, little has been written concerning the approach of bone grafting. We described a case of sacral fracture nonunion that resulted from non-operative initial treatment, which was treated successfully using bone grafting through a posterior approach and CT-guided percutaneous iliosacral screw fixation combined with anterior external fixation.

## 2. Case Presentation

**Figure 1:**
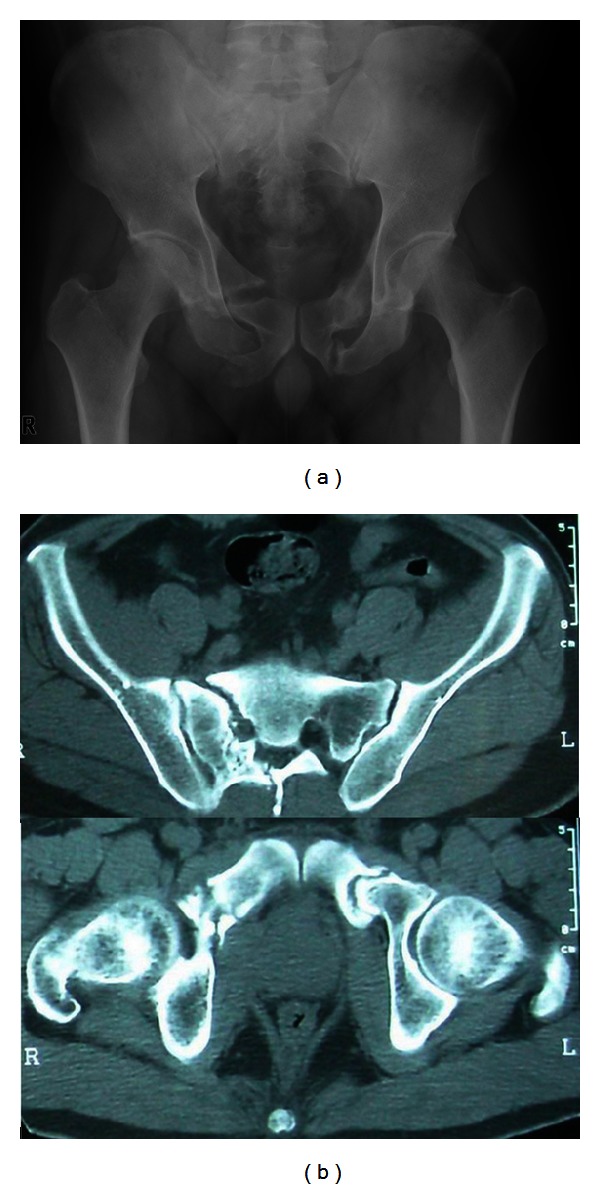
Anteroposterior radiograph (a) and CT images (b) of the pelvis at 5 months after injury, showing nonunion of the right sacrum bone and bilateral pubic rami.

A 39-year-old man was involved in a motor vehicle crash and sustained a pelvic fracture. The patient was initially treated conservatively at another hospital and 4 weeks of bed rest was advised. Six weeks after the injury, the patient started walking using double crutches. He was referred to our hospital 5 months after his initial injury with complaints of continued right posterior pelvic pain and the inability to sit comfortably. Plain pelvic radiography and CT scans revealed a fracture nonunion of the right sacrum bone and bilateral pubic rami (Figures 1(a) and 1(b)). On the initial radiograph taken right after the injury, we found a fracture of the superior and inferior pubic rami, a longitudinal fracture of the sacrum (AO/OTA classification: 61-C1) in zone II (according to Denis), and avulsion of transverse process of the L5 vertebra.

We decided to attempt a 2-step procedure. This procedure consisted of CT-guided percutaneous placement of a guide pin for a cannulated screw across the sacroiliac joint under local anesthesia in the CT suite with subsequent iliosacral screw fixation, debridement of the nonunion tissue, and bone grafting through a posterior approach, and supplementary anterior external fixation under general anesthesia after moving to the operating theater. 

**Figure 2:**
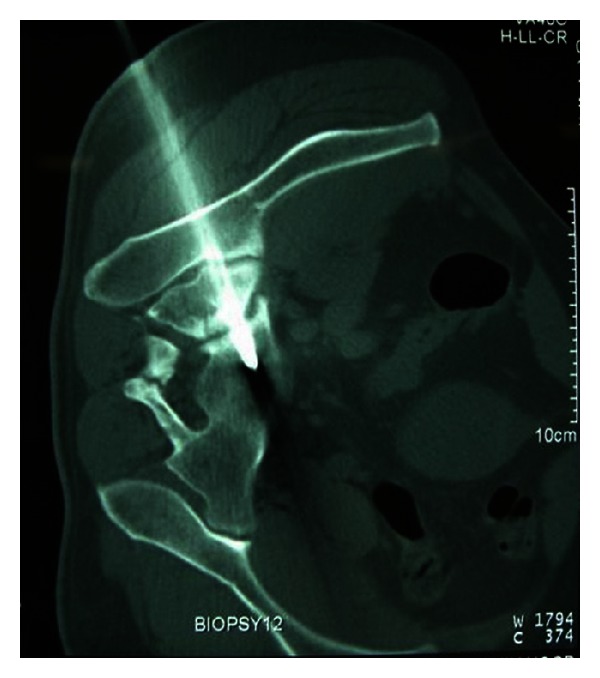
A CT image of the pelvis, showing a guide pin advanced through the iliac wing across the sacroiliac joint to the mid-body of S1.

The patient was brought to the CT suite and placed in a right lateral decubitus position on the scanning table. For intraoperative planning, a CT scan of the pelvis was obtained using a spiral CT scanner (Somatom Plus 4 Volume Zoom, Siemens AG, Forchheim, Germany; imaging parameters: 120 kV, 165 mA; slice thickness, 5 mm). Disinfection and draping were performed by an operator gowned with sterile surgical clothing. Local anesthesia consisting of 1% lidocaine was administered to the skin along the outer iliac periosteal surface. The operator made a small skin incision on the entry side and inserted a guide pin through the iliac wing across the sacroiliac joint to the mid-body of S1 using a Command 2 Console Power System (Stryker Instruments, Kalamazoo, MI). A repeated scan was taken at the level of the guide pin to check the depth, position, and angulation of the guide pin (Figure 2). After final placement of the guide pin, the patient was transferred to the operating theater with the guide pin still in place. 

**Figure 3:**
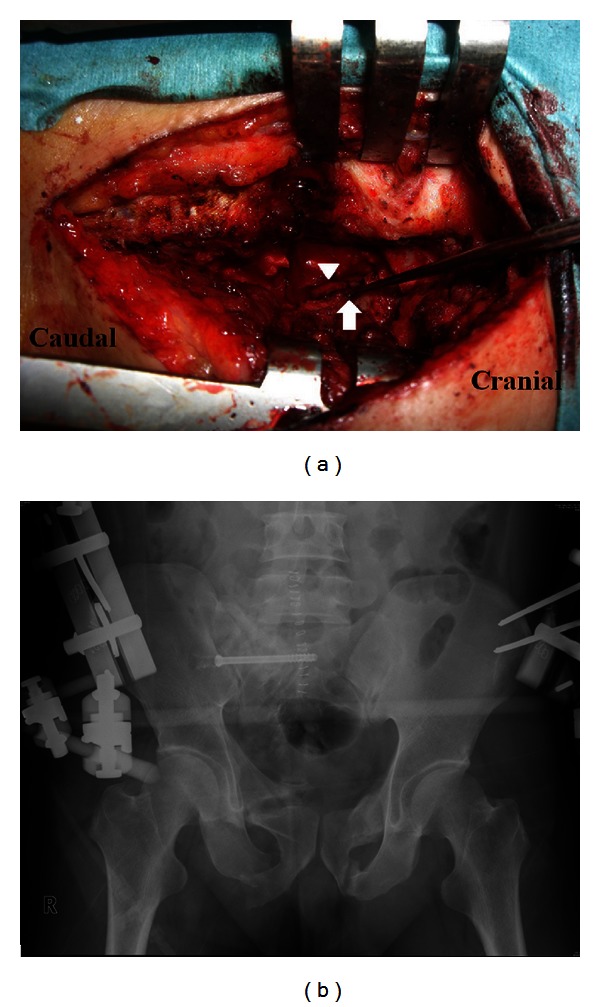
(a) Intraoperative photograph illustrating the exposure of the nonunion site. Arrowhead shows S1 nerve root. Arrow shows the nonunion site. (b) Postoperative anteroposterior radiograph.

**Figure 4:**
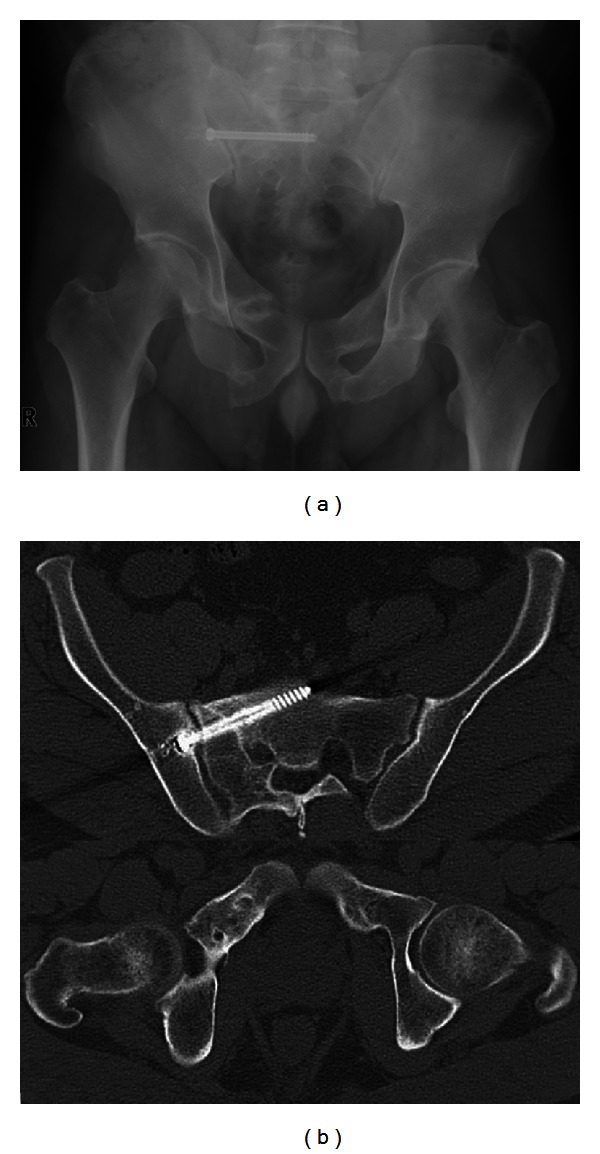
Anteroposterior radiograph (a) and (b) CT images taken 9 months after operation, which demonstrate the healing of nonunion of the right sacrum bone and bilateral superior pubic rami.

After induction of general anesthesia in the operating theater, the patient was positioned in the prone position. The screw tract was drilled over the previously placed guide pin. A 6.5 mm cannulated cancellous screw (the length was predetermined from the CT images) was then placed over the guide pin percutaneously. After the screw replacement, the right sacrum was exposed through a posterior approach. A partial hemilaminectomy of S1 and S2 was performed, the ligament flavum was excised, and the S1 nerve root was exposed (Figure 3(a)). The dural sac and S1 nerve root were retracted, and the nonunion site was exposed. We utilized the excised lamina as grafting bone. After debridement of nonunion tissue to refresh the nonunion site, the excised bone was morselized and grafted into the nonunion site. Once posterior fixation and bone grafting was accomplished, the patient was turned supine, and a supplementary anterior external fixator (Hoffmann II; Stryker, Mahwah, NJ) was applied (Figure 3(b)). The total blood loss was 60 mL. The external fixator was maintained for 4 weeks. Partial weight-bearing was permitted 4 weeks postoperatively, and full weight-bearing was permitted 3 months postoperatively. Nine months after the operation, radiographs and CT scans revealed that the fracture nonunions of the sacrum and pubic rami except the right inferior pubic rami had healed (Figures 4(a) and 4(b)). At a 2-year followup, the patient was satisfied, because he was able to walk with pain-free without aid. He was able to sit without discomfort, and had no restriction in daily activities.

## 3. Discussion

Nonunion of a sacral fracture is a rare but serious clinical condition which can cause severe chronic pain, discomfort while sitting, gait abnormalities, and significant restriction of the level of activities [4, 14]. Non-operative treatments such as bed rest, traction, and pelvic slings have been reported to be the most common initial treatment modalities leading to fracture nonunions [6, 8–14]. In the current case, despite having an unstable fracture of the pelvic ring, inadequate conservative treatment was selected as the initial treatment. Rigid internal fixation and anatomical reduction of the pelvic ring for unstable fractures has been recommended to obtain stable fixation, reduce mortality, and allow early ambulation [3, 8, 11, 16].

Surgical treatment of pelvic nonunions is technically demanding, and there are potential procedure-related complications such as nerve or vascular injuries, intraoperative blood loss, tissue loss, and infection [6, 9, 14, 16]. The gold standard treatment of fracture nonunion is a multistage approach including open excision, reaming of the nonunion site, and internal fixation [6, 9, 12–15]. Van den Bosch et al. [11] recommended the use of percutaneous iliosacral screws under fluoroscopic guidance for nonunion of unstable pelvic fractures where there is no significant leg length difference, as percutaneous iliosacral screw fixation is minimally invasive and givse good compression of the nonunion site. Percutaneous iliosacral screw fixation has been proven to be mechanically superior, when compared with external fixators, and equivalent to sacral bars and tension band plating [17]. Recently, Huegli et al. [18] reported that a nonunion of a sacral fracture was successfully treated percutaneously using CT guidance for iliosacral screw fixation. By using CT guidance, we are able to minimize risks of screw misplacement and neurological injuries.

The combination of internal fixation and bone grafting has been the preferred method for treatment of pelvic nonunion at majority of cases [6]. Autologous bone grafting can be used to fill the bony gap and enhance the healing process biologically. However, little has been written describing the approach for bone grafting. In the current case, the nonunion site in the sacrum was exposed by a partial hemilaminectomy of S1 and S2 using a posterior approach. The dural sac and S1 nerve root were carefully retracted, and the nonunion tissue was debrided to refresh the site. The excised bone obtained from the partial hemilaminectomy was then grafted into the gap. The posterior approach we utilized allowed direct visualization of the nonunion site. Visualization of the sacral foramen could preclude nerve root injury. 

Although iliosacral screws are one of the most used devices in the treatment of sacral fractures, iliosacral screw fixation alone may not be sufficient to achieve the stability in some unstable ring fractures [19, 20]. Routt Jr. and Simonian [17] reported a 13% failure rate of iliosacral screw fixation for sacral fractures. In our case, since anterior ring instability also existed because of the nonunion of bilateral pubic rami, we added anterior external fixation to supplement whole pelvic ring stability. Consequently, nonunion of both the superior pubic rami and the sacrum healed.

In conclusion, by CT-guided percutaneous iliosacral screw fixation and bone grafting through a posterior approach combined with anterior external fixation, we were able to obtain the healing of fracture nonunion of the sacrum and pubic rami, leading to pain relief and functional recovery.
